# Supplementary material for: Effects of Wearing FFP2 Masks on SARS-CoV-2 Infection Rates in Classrooms
Source: Int J Environ Res Public Health. 2022 Oct 19;19(20):13511. doi: 10.3390/ijerph192013511 (PMC9603337; doi:10.3390/ijerph192013511)
Supplement: Supplementary file 1 [file ijerph-19-13511-s001.zip › ijerph-1927122-supplementary.pdf]

Supplementary Online Content

Effects of wearing FFP2 masks on SARS-CoV-2 infection rates in classrooms

Jarnig G, Kerbl R, van Poppel MNM, et al.

Table S1. Description of the 4 time periods within the course of the study.
Table S2. Sample characteristics for total study population and subgroups middle and high school
Table S3. Cumulative percentage of students with a SARS-CoV-2 infection: students in classes with GB vs students in SF classes for subgroups sex.

Table S4. Detailed increase of cumulative numbers and percentage of students with a SARS-CoV-2 infection for P1 (Sep.21 and Oct.21).
Table S5. Detailed increase of cumulative numbers and percentage of students with a SARS-CoV-2 infection for P2 (Nov.21 and Dec.21).
Table S6. Detailed increase of cumulative numbers and percentage of students with a SARS-CoV-2 infection for P3 (Jan.22 and Feb.22).
Table S7. Detailed increase of cumulative numbers and percentage of students with a SARS-CoV-2 infection for P4 (Mar.22 and Apr.22).
Table S8. Detailed 7-day incidences for detected SARS-CoV-2 infections for P1 (Sep.21 and Oct.21).
Table S9. Detailed 7-day incidences for detected SARS-CoV-2 infections for P2 (Nov.21 and Dec.21).
Table S10. Detailed 7-day incidences for detected SARS-CoV-2 infections for P3 (Jan.22 and Feb.22).
Table S11. Detailed 7-day incidences for detected SARS-CoV-2 infections for P4 (Mar.22 and Apr.22).
Table S12. Potential SARS-CoV-2 infection in school classes GB vs. SF classes, using hypothesized generation time - 2 days.

Table S13. Potential SARS-CoV-2 infection in school classes GB vs. SF classes, using hypothesized generation time - 4 days.

Table S14. Potential SARS-CoV-2 infection in school classes GB vs. SF classes, using hypothesized generation time - 6 days.

Table S15. Potential SARS-CoV-2 infection in school classes GB vs. SF classes, using hypothesized generation time - 8 days.

Table S16. Binary logistic regression for Potential SARS-Cov-2 contagions in the classroom and generation times 4 and 6, for subgroup sex .

Table S17. Binary logistic regression for Potential SARS-Cov-2 contagions in the classroom and generation times 2 and 8, for subgroup sex .

Figure S1. Cumulative percentage of children with SARS-CoV-2 infections, for subgroup sex.

This supplementary material has been provided by the authors to give readers additional information about their work.

Table S1. Description of the 4 time periods within the course of the study.

Time period	date range	Mitigation measures at school not included in physical education classes		School sports		Predominant SARS-CoV-2 variant	AGES	
		GB	SF	GB	SF		Mean 7-day I (5 to 14)	Mean 7-day I (15 to 24)
P1	September 13, 2021 to October 31,	A, B	A, B	Outdoor without restrictions, indoor with restrictions	No restrictions	B.1.617.2	370.02	268.88
P2	November 1, 2021 to December 31,	C	C	Outdoor with restrictions, indoor with strict restrictions and wearing FFP2 masks	No restrictions	B.1.617.2	1521.82	962.70
P3	January 1, 2022 to February 28, 2022	C	C	Outdoor with restrictions, indoor with strict restrictions and wearing FFP2 masks	No restrictions	BA.1	2797.19	2497.02
P4	March 1, 2022 to April 30, 2022	D	D	Outdoor without restrictions, indoor with restrictions	No restrictions	BA.2/BA.3	2361.77	2185.20

SARS-CoV-2 = severe acute respiratory syndrome coronavirus 2, GB = students in school classes with a general school branch, SF = students in school classes with sport focus, AGES = Austrian Agency for Health and Food Safety, 7-day I (5 to 14) = 7 days incidence for SARS-CoV-2 infections per 100,000 inhabitants in Carinthia (AUT) for children aged 5 to 14 years (https://covid19-dashboard.ages.at/), 7-day I (15 to 24) = 7 days incidence for SARS-CoV-2 infections per 100,000 inhabitants in Carinthia (AUT) for adolescent aged 15 to 24 years (https://covid19-dashboard.ages.at/), AUT = Austria, P1 = period 1 (September 13, 2021 to October 31, 2021) P2 = period 2 (November 1, 2021 to December 31, 2021), P3 = period 3 (January 1, 2022 to February 28, 2022), P4 = period 4 (March 1, 2022 to April 30, 2022), A = Risk level 1: All students can be tested voluntarily at school for SARS-CoV-2 infection by rapid anterionasal antigen test, B = Risk level 2: Unvaccinated students are tested three times weekly for SARS-CoV-2 infection (twice anterionasal rapid antigen test, once PCR test with mouth rinse) and Students are required to wear a face mask outside of classrooms, PCR = polymerase chain reaction, C = All students are tested three times weekly for SARS-CoV-2 infection (once anterionasal rapid antigen test, twice PCR test with mouth rinse) and Students are required to wear a FFP2 face mask anywhere in school building, FFP2 = filtering face piece generation 2 (FFP2 masks: have a protective effect of > 95 % for airborne particles in controlled laboratory tests; air permeability and airway resistance are standardized. The leakage is < 11 %), D = All students are tested three times weekly for SARS-CoV-2 infection (once anterionasal rapid antigen test, twice PCR test with mouth rinse), B.1.617.2 = Delta variant of SARS-CoV-2 Virus, BA.1 = Omicron variant of SARS-CoV-2 Virus, BA.2/BA.3 = Omicron variant of SARS-CoV-2 Virus.

Table S2. Sample characteristics for total study population and subgroups middle and high school

Variable	All (n=614)	GB (n=419)	SF (n=195)	X^2	t	*P* value	p-lvl
Female, No. (%)	265 (43.2%)	216 (51.6%)	49 (25.1%)	37.872		<.001	***
Age at index, mean (SD)	15.1 ± 2.3	15.3 ± 2.3	14.8 ± 2.2		2.752	0.006	**
SARS-CoV-2 infection - bevor September 13, 2021, No. (%)	76 (12.4%)	44 (10.5%)	32 (16.4%)	4.284		0.038	*
SARS-CoV-2 infection - September 13, 2021 to April 30, 2022, No. (%)	353 (57.5%)	229 (54.7%)	124 (63.6%)	4.348		0.037	*
	M.S.						
	All (n=289)	GB (n=190)	SF (n=99)				
Female, No. (%)	111 (38.4%)	84 (44.2%)	27 (27.3%)	7.893		0.005	**
Age at index, mean (SD)	13.1 ± 1.3	13.2 ± 1.3	12.9 ± 1.2		2.105	0.036	*
SARS-CoV-2 infection - bevor September 13, 2021, No. (%)	27 (9.3%)	17 (8.9%)	10 (10.1%)	0.102		0.75	
SARS-CoV-2 infection - September 13, 2021 to April 30, 2022, No. (%)	173 (59.9%)	107 (56.3%)	66 (66.7%)	2.902		0.09	
	H.S.						
	All (n=325)	GB (n=229)	SF (n=96)				
Female, No. (%)	154 (47.4%)	132 (57.6%)	22 (22.9%)	32.716		<.001	***
Age at index, mean (SD)	16.9 ± 1.2	17.0 ± 1.3	16.7 ± 1.2		2.258	0.025	*
SARS-CoV-2 infection - bevor September 13, 2021, No. (%)	49 (15.1%)	27 (11.8%)	22 (22.9%)	6.540		0.011	*
SARS-CoV-2 infection - September 13, 2021 to April 30, 2022, No. (%)	180 (55.4%)	122 (53.3%)	58 (60.4%)	1.396		0.24	

SARS-CoV-2 = severe acute respiratory syndrome coronavirus 2, Data are absolute numbers (No.) and percentages (%), X^2 = Chi-Square Test value, t = test statistic t-test, p-lvl (P Value level) * = P < 0.05, ** = P < 0.01, *** = P < 0.001, p-lvl = significance level, No. = Number, % = Percentage, M.S. = middle school (Students aged 13.1 ± 1.3 years old), H.S. = 4-year high school (Students aged 16.9 ± 1.2 years old), GB = students in school classes with a general school branch, SF = students in school classes with sport focus, n = Study population.

Table S3. Cumulative percentage of students with a SARS-CoV-2 infection: students in classes with GB vs students in SF classes for subgroups sex.

Variable	Time period	Boys							Girls						
		All (n=349)		X^2	p Value [b]	p-lvl	φ	OR (95% CI)	All (n=265)		X^2	p Value [b]	p-lvl	φ	OR (95% CI)
		GB (n=203)	SF (n=146)						GB (n=216)	SF (n=49)					
CU% SARS-CoV-2 …, No. [%]	…Sep.21	1 (0.5%)	2 (1.4%)	0.767 [a]	0.57		0.05	2.81 (0.25 to 31.24)	0 (0.0%)	0 (0.0%)	INC				
	…Oct.21	3 (1.5%)	5 (3.4%)	1.437 [a]	0.29		0.06	2.36 (0.56 to 10.05)	2 (0.9%)	0 (0.0%)	0.457 [a]	>.99		-0.04	0.81 (0.77 to 0.86)
	…Nov.21	10 (4.9%)	23 (15.8%)	11.628	0.001	**	0.18	3.61 (1.66 to 7.84)	16 (7.4%)	6 (12.2%)	1.228 [a]	0.26		0.07	1.74 (0.65 to 4.72)
	…Dec.21	15 (7.4%)	25 (17.1%)	7.930	0.005	**	0.15	2.59 (1.31 to 5.11)	23 (10.6%)	7 (14.3%)	0.526	0.47		0.05	1.40 (0.56 to 3.47)
	…Jan.22	35 (17.2%)	56 (38.4%)	19.642	<.001	***	0.24	2.99 (1.82 to 4.89)	46 (21.3%)	29 (59.2%)	28.253	<.001	***	0.33	5.36 (2.78 to 10.33)
	…Feb.22	66 (32.5%)	83 (56.8%)	20.559	<.001	***	0.24	2.74 (1.76 to 4.25)	83 (38.4%)	32 (65.3%)	11.748	0.001	**	0.21	3.02 (1.58 to 5.77)
	…Mar.22	98 (48.3%)	87 (59.6%)	4.363	0.037	*	0.11	1.58 (1.03 to 2.43)	117 (54.2%)	33 (67.3%)	2.825	0.09		0.10	1.75 (0.91 to 3.36)
	…Apr.22	104 (51.2%)	91 (62.3%)	4.242	0.039	*	0.11	1.58 (1.02 to 2.43)	125 (57.9%)	33 (67.3%)	1.49	0.22		0.08	1.45 (1.02 to 2.06)
		M.S. (n=178)							M.S. (n=111)						
		GB (n=106)	SF (n=72)						GB (n=84)	SF (n=27)					
CU% SARS-CoV-2 …, No. [%]	…Sep.21	0 (0.0%)	1 (1.4%)	1.481 [a]	0.40		0.09	0.40 (0.34 to 0.48)	0 (0.0%)	0 (0.0%)	INC				
	…Oct.21	1 (0.9%)	2 (2.8%)	0.871 [a]	0.57		0.07	3.00 (0.27 to 33.72)	1 (1.2%)	0 (0.0%)	0.324 [a]	>.99		-0.05	0.76 (0.68 to 0.84)
	…Nov.21	5 (4.7%)	12 (16.7%)	7.088	0.008	**	0.20	4.04 (1.36 to 12.03)	4 (4.8%)	3 (11.1%)	1.394 a	0.36		0.11	2.50 (0.52 to 11.96)
	…Dec.21	8 (7.5%)	14 (19.4%)	5.603	0.018	*	0.18	2.96 (1.17 to 7.48)	7 (8.3%)	4 (14.8%)	0.961 a	0.46		0.09	1.91 (0.51 to 7.12)
	…Jan.22	17 (16.0%)	23 (31.9%)	6.227	0.013	*	0.19	2.46 (1.20 to 5.04)	16 (19.0%)	17 (63.0%)	18.862	<.001	***	0.41	7.23 (2.79 to 18.72)
	…Feb.22	39 (36.8%)	40 (55.6%)	6.115	0.013	*	0.19	2.15 (1.17 to 3.95)	31 (36.9%)	20 (74.1%)	11.366	0.001	**	0.32	4.89 (1.86 to 12.86)
	…Mar.22	55 (51.9%)	43 (59.7%)	1.064	0.30		0.08	1.38 (0.75 to 2.52)	46 (54.8%)	21 (77.8%)	4.524	0.033	*	0.20	2.89 (1.06 to 7.89)
	…Apr.22	57 (53.8%)	45 (62.5%)	1.334	0.25		0.09	1.43 (0.78 to 2.64)	50 (59.5%)	21 (77.8%)	2.954	0.09		0.16	2.38 (0.87 to 6.51)
		H.S. (n=171)							H.S. (n=154)						
		GB (n=97)	SF (n=74)						GB (n=132)	SF (n=22)					
CU% SARS-CoV-2 …, No. [%]	…Sep.21	1 (1.0%)	1 (1.4%)	0.037[a]	>.99		0.02	1.32 (0.08 to 21.38)	0 (0.0%)	0 (0.0%)	INC				
	…Oct.21	2 (2.1%)	3 (4.1%)	0.587[a]	0.65		0.06	2.00 (0.33 to 12.33)	1 (1.2%)	0 (0.0%)	0.168 [a]	>.99		-0.03	0.86 (0.80 to 0.91)
	…Nov.21	5 (5.2%)	11 (14.9%)	4.667	0.031	*	0.17	3.21 (1.06 to 9.70)	12 (9.1%)	3 (13.6%)	0.443 [a]	0.45		0.05	1.58 (0.41 to 6.12)
	…Dec.21	7 (7.2%)	11 (14.9%)	2.607	0.11		0.12	2.25 (0.83 to 6.11)	16 (12.1%)	3 (13.6%)	0.040 [a]	0.74		0.02	1.15 (0.30 to 4.31)
	…Jan.22	18 (18.6%)	33 (44.6%)	13.598	<.001	***	0.28	3.53 (1.78 to 7.02)	30 (22.7%)	12 (54.5%)	9.625	0.002	**	0.25	4.08 (1.61 to 10.37)
	…Feb.22	27 (27.8%)	43 (58.1%)	15.911	<.001	***	0.31	3.60 (1.90 to 6.82)	52 (39.4%)	12 (54.5%)	1.782	0.18		0.11	1.85 (0.74 to 4.58)
	…Mar.22	43 (44.3%)	44 (59.5%)	3.845	0.05	*	0.15	1.84 (1.00 to 3.40)	71 (53.8%)	12 (54.5%)	0.004	0.95		0.01	1.03 (0.42 to 2.55)
	…Apr.22	47 (48.5%)	46 (62.2%)	3.180	0.08		0.14	1.75 (0.94 to 3.24)	75 (56.8%)	12 (54.5%)	0.040	0.84		-0.02	0.91 (0.37 to 2.26)

a = 2 cells (50,0%) have expected count less than 5; b = If (a) occurred, Fisher's Exact Test was used. Data are No. (%), n = Study population, GB = students in school classes with a general school branch, SF = students in school classes with sport focus, X^2 = Chi-Square Test value, p-lvl (P Value level) * = P < 0.05, ** = P < 0.01, *** = P < 0.001, p-lvl = significance level, φ = Effect size Phi, OR = odds ratio (with 1 for students in classes without sport focus), CI = confidence interval, CU% SARS-CoV-2 = cumulative percentage of students with a SARS-COV-2 infections at the End of…, SARS-CoV-2 = severe acute respiratory syndrome coronavirus 2, P1 = period 1 (September 13, 2021 to October 31, 2021) P2 = period 2 (November 1, 2021 to December 31, 2021), P3 = period 3 (January 1, 2022 to February 28, 2022), P4 = period 4 (March 1, 2022 to April 30, 2022), INC = insufficient number of potential infection cases.

Table S4. Detailed increase of cumulative numbers and percentage of students with a SARS-CoV-2 infection for P1 (Sep.21 and Oct.21).

Mo.	M.S.				H.S.				Mo.	M.S.				H.S.			
Sep.21	GB (n=190)		SF (n=99)		GB (n=229)		SF (n=96)		Oct.21	GB (n=190)		SF (n=99)		GB (n=229)		SF (n=96)	
	No.	%	No.	%	No.	%	No.	%		No.	%	No.	%	No.	%	No.	%
1									1	0	0.00%	1	1.00%	1	0.40%	1	1.00%
2									2	0	0.00%	1	1.00%	1	0.40%	1	1.00%
3									3	0	0.00%	1	1.00%	1	0.40%	1	1.00%
4									4	0	0.00%	1	1.00%	1	0.40%	1	1.00%
5									5	0	0.00%	1	1.00%	2	0.90%	1	1.00%
6									6	0	0.00%	1	1.00%	2	0.90%	1	1.00%
7									7	0	0.00%	1	1.00%	2	0.90%	1	1.00%
8									8	0	0.00%	1	1.00%	2	0.90%	1	1.00%
9									9	0	0.00%	1	1.00%	2	0.90%	1	1.00%
10									10	0	0.00%	1	1.00%	2	0.90%	1	1.00%
11									11	0	0.00%	1	1.00%	2	0.90%	1	1.00%
12									12	0	0.00%	1	1.00%	2	0.90%	1	1.00%
13	0	0.00%	0	0.00%	0	0.00%	0	0.00%	13	0	0.00%	1	1.00%	2	0.90%	1	1.00%
14	0	0.00%	0	0.00%	0	0.00%	0	0.00%	14	0	0.00%	1	1.00%	2	0.90%	2	2.10%
15	0	0.00%	0	0.00%	0	0.00%	0	0.00%	15	0	0.00%	1	1.00%	2	0.90%	2	2.10%
16	0	0.00%	0	0.00%	0	0.00%	0	0.00%	16	0	0.00%	1	1.00%	2	0.90%	2	2.10%
17	0	0.00%	0	0.00%	0	0.00%	0	0.00%	17	0	0.00%	1	1.00%	2	0.90%	2	2.10%
18	0	0.00%	0	0.00%	0	0.00%	0	0.00%	18	0	0.00%	1	1.00%	2	0.90%	2	2.10%
19	0	0.00%	0	0.00%	0	0.00%	0	0.00%	19	0	0.00%	1	1.00%	2	0.90%	2	2.10%
20	0	0.00%	0	0.00%	0	0.00%	0	0.00%	20	1	0.50%	2	2.00%	2	0.90%	2	2.10%
21	0	0.00%	0	0.00%	0	0.00%	0	0.00%	21	1	0.50%	2	2.00%	2	0.90%	2	2.10%
22	0	0.00%	0	0.00%	0	0.00%	0	0.00%	22	2	1.10%	2	2.00%	2	0.90%	2	2.10%
23	0	0.00%	0	0.00%	0	0.00%	1	1.00%	23	2	1.10%	2	2.00%	2	0.90%	2	2.10%
24	0	0.00%	0	0.00%	0	0.00%	1	1.00%	24	2	1.10%	2	2.00%	2	0.90%	2	2.10%
25	0	0.00%	0	0.00%	0	0.00%	1	1.00%	25	2	1.10%	2	2.00%	2	0.90%	2	2.10%
26	0	0.00%	0	0.00%	0	0.00%	1	1.00%	26	2	1.10%	2	2.00%	2	0.90%	2	2.10%
27	0	0.00%	0	0.00%	1	0.40%	1	1.00%	27	2	1.10%	2	2.00%	2	0.90%	3	3.10%
28	0	0.00%	0	0.00%	1	0.40%	1	1.00%	28	2	1.10%	2	2.00%	3	1.30%	3	3.10%
29	0	0.00%	1	1.00%	1	0.40%	1	1.00%	29	2	1.10%	2	2.00%	3	1.30%	3	3.10%
30	0	0.00%	1	1.00%	1	0.40%	1	1.00%	30	2	1.10%	2	2.00%	3	1.30%	3	3.10%
									31	2	1.10%	2	2.00%	3	1.30%	3	3.10%

SARS-CoV-2 = severe acute respiratory syndrome coronavirus 2, P1 = period 1 (September 13, 2021 to October 31, 2021), Data are cumulative numbers (No.) and percentages (%) of SARS-CoV-2 infections detected, No. = Number, % = Percentage, Mo. = month, M.S. = middle school (Students aged 13.1 ± 1.3 years old), H.S. = 4-year high school (Students aged 16.9 ± 1.2 years old), GB = students in school classes with a general school branch, SF = students in school classes with sport focus, n = Study population, Sep.21 = September 2021, Oct.21 = October 2021.

Table S5. Detailed increase of cumulative numbers and percentage of students with a SARS-CoV-2 infection for P2 (Nov.21 and Dec.21).

Mo.	M.S.				H.S.				Mo.	M.S.				H.S.			
Nov.21	GB (n=190)		SF (n=99)		GB (n=229)		SF (n=96)		Dec.21	GB (n=190)		SF (n=99)		GB (n=229)		SF (n=96)	
	No.	%	No.	%	No.	%	No.	%		No.	%	No.	%	No.	%	No.	%
1	2	1.1%	3	3.0%	3	1.3%	3	3.1%	1	10	5.3%	15	15.2%	17	7.4%	14	14.6%
2	2	1.1%	4	4.0%	3	1.3%	3	3.1%	2	10	5.3%	15	15.2%	17	7.4%	14	14.6%
3	2	1.1%	5	5.1%	3	1.3%	4	4.2%	3	10	5.3%	15	15.2%	17	7.4%	14	14.6%
4	3	1.6%	5	5.1%	4	1.7%	4	4.2%	4	10	5.3%	15	15.2%	17	7.4%	14	14.6%
5	3	1.6%	5	5.1%	6	2.6%	4	4.2%	5	10	5.3%	15	15.2%	18	7.9%	14	14.6%
6	3	1.6%	5	5.1%	6	2.6%	5	5.2%	6	11	5.8%	16	16.2	19	8.3%	14	14.6%
7	3	1.6%	5	5.1%	7	3.1%	5	5.2%	7	11	5.8%	16	16.2%	20	8.7%	14	14.6%
8	3	1.6%	6	6.1%	8	3.5%	7	7.3%	8	11	5.8%	16	16.2%	20	8.7%	14	14.6%
9	3	1.6%	6	6.1%	8	3.5%	8	8.3%	9	11	5.8%	16	16.2%	20	8.7%	14	14.6%
10	3	1.6%	6	6.1%	9	3.9%	9	9.4%	10	12	6.3%	16	16.2%	21	9.2%	14	14.6%
11	3	1.6%	6	6.1%	11	4.8%	9	9.4%	11	13	6.8%	16	16.2%	21	9.2%	14	14.6%
12	4	2.1%	6	6.1%	11	4.8%	9	9.4%	12	13	6.8%	16	16.2%	21	9.2%	14	14.6%
13	5	2.6%	6	6.1%	11	4.8%	9	9.4%	13	14	7.4%	16	16.2%	23	10.0%	14	14.6%
14	5	2.6%	6	6.1%	12	5.2%	9	9.4%	14	14	7.4%	16	16.2%	23	10.0%	14	14.6%
15	6	3.2%	6	6.1%	13	5.7%	10	10.4%	15	15	7.9%	16	16.2%	23	10.0%	14	14.6%
16	6	3.2%	7	7.1%	13	5.7%	10	10.4%	16	15	7.9%	16	16.2%	23	10.0%	14	14.6%
17	6	3.2%	7	7.1%	14	6.1%	10	10.4%	17	15	7.9%	16	16.2%	23	10.0%	14	14.6%
18	6	3.2%	7	7.1%	15	6.6%	10	10.4%	18	15	7.9%	16	16.2%	23	10.0%	14	14.6%
19	6	3.2%	7	7.1%	16	7.0%	11	11.5%	19	15	7.9%	16	16.2%	23	10.0%	14	14.6%
20	6	3.2%	7	7.1%	16	7.0%	13	13.5%	20	15	7.9%	16	16.2%	23	10.0%	14	14.6%
21	6	3.2%	7	7.1%	16	7.0%	13	13.5%	21	15	7.9%	16	16.2	23	10.0%	14	14.6%
22	6	3.2%	7	7.1%	16	7.0%	13	13.5%	22	15	7.9%	16	16.2%	23	10.0%	14	14.6%
23	6	3.2%	7	7.1%	16	7.0%	13	13.5%	23	15	7.9%	16	16.2%	23	10.0%	14	14.6%
24	6	3.2%	9	9.1%	16	7.0%	13	13.5%	24	15	7.9%	16	16.2%	23	10.0%	14	14.6%
25	7	3.7%	12	12.1%	16	7.0%	13	13.5%	25	15	7.9%	16	16.2%	23	10.0%	14	14.6%
26	8	4.2%	14	14.1%	17	7.4%	14	14.6%	26	15	7.9%	17	17.2%	23	10.0%	14	14.6%
27	8	4.2%	14	14.1%	17	7.4%	14	14.6%	27	15	7.9%	17	17.2%	23	10.0%	14	14.6%
28	8	4.2%	14	14.1%	17	7.4%	14	14.6%	28	15	7.9%	17	17.2%	23	10.0%	14	14.6%
29	8	4.2%	15	15.2%	17	7.4%	14	14.6%	29	15	7.9%	18	18.2%	23	10.0%	14	14.6%
30	9	4.7%	15	15.2%	17	7.4%	14	14.6%	30	15	7.9%	18	18.2%	23	10.0%	14	14.6%
									31	15	7.9%	18	18.2%	23	10.0%	14	14.6%

SARS-CoV-2 = severe acute respiratory syndrome coronavirus 2, P2 = period 2 (November 1, 2021 to December 31, 2021), Data are cumulative numbers (No.) and percentages (%) of SARS-CoV-2 infections detected, No. = Number, % = Percentage, Mo. = month, M.S. = middle school (Students aged 13.1 ± 1.3 years old), H.S. = 4-year high school (Students aged 16.9 ± 1.2 years old), GB = students in school classes with a general school branch, SF = students in school classes with sport focus, n = Study population, Nov.21 = November 2021, Dec.21 = December 2021.

Table S6. Detailed increase of cumulative numbers and percentage of students with a SARS-CoV-2 infection for P3 (Jan.22 and Feb.22).

Mo.	M.S.				H.S.				Mo.	M.S.				H.S.			
Jan.22	GB (n=190)		SF (n=99)		GB (n=229)		SF (n=96)		Feb.22	GB (n=190)		SF (n=99)		GB (n=229)		SF (n=96)	
	No.	%	No.	%	No.	%	No.	%		No.	%	No.	%	No.	%	No.	%
1	15	7.9%	18	18.2%	24	10.5%	14	14.6%	1	36	18.9%	40	40.4%	50	21.8%	46	47.9%
2	15	7.9%	18	18.2%	24	10.5%	14	14.6%	2	37	19.5%	44	44.4%	51	22.3%	46	47.9%
3	15	7.9%	18	18.2%	24	10.5%	14	14.6%	3	39	20.5%	44	44.4%	51	22.3%	48	50.0%
4	15	7.9%	18	18.2%	24	10.5%	15	15.6%	4	41	21.6%	45	45.5%	51	22.3%	49	51.0%
5	16	8.4%	18	18.2%	24	10.5%	15	15.6%	5	42	22.1%	46	46.5%	52	22.7%	50	52.1%
6	16	8.4%	18	18.2%	24	10.5%	16	16.7%	6	42	22.1%	47	47.5%	52	22.7%	52	54.2%
7	16	8.4%	19	19.2%	25	10.9%	16	16.7%	7	42	22.1%	48	48.5%	54	23.6%	52	54.2%
8	16	8.4%	19	19.2%	26	11.4%	17	17.7%	8	43	22.6%	51	51.5%	55	24.0%	53	55.2%
9	17	8.9%	20	20.2%	27	11.8%	17	17.7%	9	45	23.7%	52	52.5%	57	24.9%	53	55.2%
10	21	11.1%	21	21.2%	27	11.8%	18	18.8%	10	47	24.7%	55	55.6%	58	25.3%	54	56.3%
11	23	12.1%	22	22.2%	27	11.8%	19	19.8%	11	47	24.7%	55	55.6%	58	25.3%	54	56.3%
12	23	12.1%	22	22.2%	27	11.8%	20	20.8%	12	49	25.8%	55	55.6%	58	25.3%	54	56.3%
13	24	12.6%	22	22.2%	27	11.8%	20	20.8%	13	51	26.8%	55	55.6%	59	25.8%	54	56.3%
14	24	12.6%	22	22.2%	27	11.8%	20	20.8%	14	52	27.4%	55	55.6%	61	26.6%	54	56.3%
15	24	12.6%	22	22.2%	30	13.1%	20	20.8%	15	52	27.4%	56	56.6%	66	28.8%	54	56.3%
16	24	12.6%	22	22.2%	30	13.1%	22	22.9%	16	52	27.4%	56	56.6%	66	28.8%	54	56.3%
17	24	12.6%	23	23.2%	33	14.4%	23	24.0%	17	55	28.9%	56	56.6%	66	28.8%	54	56.3%
18	25	13.2%	24	24.2%	34	14.8%	27	28.1%	18	56	29.5%	57	57.6%	68	29.7%	54	56.3%
19	26	13.7%	24	24.2%	37	16.2%	29	30.2%	19	59	31.1%	57	57.6%	68	29.7%	54	56.3%
20	26	13.7%	25	25.3%	39	17.0%	31	32.3%	20	63	33.2%	57	57.6%	69	30.1%	54	56.3%
21	27	14.2%	27	27.3%	40	17.5%	34	35.4%	21	65	34.2%	58	58.6%	71	31.0%	54	56.3%
22	28	14.7%	28	28.3%	40	17.5%	39	40.6%	22	67	35.3%	58	58.6%	74	32.3%	55	57.3%
23	29	15.3%	29	29.3%	40	17.5%	40	41.7%	23	67	35.3%	59	59.6%	77	33.6%	55	57.3%
24	29	15.3%	30	30.3%	40	17.5%	41	42.7%	24	67	35.3%	59	59.6%	78	34.1%	55	57.3%
25	30	15.8%	30	30.3%	41	17.9%	42	43.8%	25	69	36.3%	59	59.6%	78	34.1%	55	57.3%
26	30	15.8%	34	34.3%	41	17.9%	42	43.8%	26	69	36.3%	60	60.6%	78	34.1%	55	57.3%
27	31	16.3%	37	37.4%	42	18.3%	44	45.8%	27	69	36.3%	60	60.6%	78	34.1%	55	57.3%
28	32	16.8%	38	38.4%	42	18.3%	44	45.8%	28	70	36.8%	60	60.6%	79	34.5%	55	57.3%
29	32	16.8%	38	38.4%	43	18.8%	44	45.8%									
30	32	16.8%	38	38.4%	46	20.1%	44	45.8%									
31	33	17.4%	40	40.4%	48	21.0%	45	46.9%									

SARS-CoV-2 = severe acute respiratory syndrome coronavirus 2, P3 = period 3 (January 1, 2022 to February 28, 2022), Data are cumulative numbers (No.) and percentages (%) of SARS-CoV-2 infections detected, No. = Number, % = Percentage, Mo. = month, M.S. = middle school (Students aged 13.1 ± 1.3 years old), H.S. = 4-year high school (Students aged 16.9 ± 1.2 years old), GB = students in school classes with a general school branch, SF = students in school classes with sport focus, n = Study population, Jan.22 = January 2022, Feb.22 = February 2022.

Table S7. Detailed increase of cumulative numbers and percentage of students with a SARS-CoV-2 infection for P4 (Mar.22 and Apr.22).

Mo.	M.S.				H.S.				Mo.	M.S.				H.S.			
Mar.22	GB (n=190)		SF (n=99)		GB (n=229)		SF (n=96)		Apr.22	GB (n=190)		SF (n=99)		GB (n=229)		SF (n=96)	
	No.	%	No.	%	No.	%	No.	%		No.	%	No.	%	No.	%	No.	%
1	70	36.8%	61	61.6%	82	35.8%	55	57.3%	1	101	53.2%	64	64.6%	115	50.2%	56	58.3%
2	72	37.9%	61	61.6%	83	36.2%	55	57.3%	2	102	53.7%	64	64.6%	115	50.2%	56	58.3%
3	73	38.4%	61	61.6%	83	36.2%	55	57.3%	3	105	55.3%	64	64.6%	115	50.2%	56	58.3%
4	73	38.4%	62	62.6%	84	36.7%	55	57.3%	4	105	55.3%	65	65.7%	115	50.2%	56	58.3%
5	73	38.4%	62	62.6%	86	37.6%	55	57.3%	5	105	55.3%	65	65.7%	115	50.2%	56	58.3%
6	73	38.4%	62	62.6%	86	37.6%	55	57.3%	6	105	55.3%	65	65.7%	116	50.7%	56	58.3%
7	73	38.4%	62	62.6%	87	38.0%	56	58.3%	7	105	55.3%	65	65.7%	117	51.1%	56	58.3%
8	74	38.9%	62	62.6%	87	38.0%	56	58.3%	8	105	55.3%	65	65.7%	118	51.5%	56	58.3%
9	78	41.1%	62	62.6%	90	39.3%	56	58.3%	9	105	55.3%	65	65.7%	118	51.5%	56	58.3%
10	80	42.1%	62	62.6%	93	40.6%	56	58.3%	10	106	55.8%	65	65.7%	118	51.5%	56	58.3%
11	83	43.7%	62	62.6%	93	40.6%	56	58.3%	11	106	55.8%	65	65.7%	118	51.5%	56	58.3%
12	86	45.3%	62	62.6%	93	40.6%	56	58.3%	12	106	55.8%	65	65.7%	118	51.5%	56	58.3%
13	87	45.8%	62	62.6%	93	40.6%	56	58.3%	13	106	55.8%	65	65.7%	118	51.5%	56	58.3%
14	89	46.8%	62	62.6%	93	40.6%	56	58.3%	14	106	55.8%	65	65.7%	119	52.0%	56	58.3%
15	91	47.9%	62	62.6%	94	41.0%	56	58.3%	15	107	56.3%	65	65.7%	119	52.0%	56	58.3%
16	95	50.0%	62	62.6%	98	42.8%	56	58.3%	16	107	56.3%	65	65.7%	119	52.0%	56	58.3%
17	96	50.5%	62	62.6%	99	43.2%	56	58.3%	17	107	56.3%	65	65.7%	119	52.0%	57	59.4%
18	96	50.5%	62	62.6%	101	44.1%	56	58.3%	18	107	56.3%	65	65.7%	119	52.0%	57	59.4%
19	96	50.5%	62	62.6%	101	44.1%	56	58.3%	19	107	56.3%	65	65.7%	120	52.4%	57	59.4%
20	96	50.5%	62	62.6%	105	45.9%	56	58.3%	20	107	56.3%	65	65.7%	121	52.8%	57	59.4%
21	97	51.1%	62	62.6%	107	46.7%	56	58.3%	21	107	56.3%	65	65.7%	121	52.8%	57	59.4%
22	98	51.6%	62	62.6%	110	48.0%	56	58.3%	22	107	56.3%	65	65.7%	121	52.8%	57	59.4%
23	98	51.6%	63	63.6%	112	48.9%	56	58.3%	23	107	56.3%	65	65.7%	121	52.8%	57	59.4%
24	99	52.1%	63	63.6%	112	48.9%	56	58.3%	24	107	56.3%	65	65.7%	121	52.8%	57	59.4%
25	99	52.1%	63	63.6%	112	48.9%	56	58.3%	25	107	56.3%	65	65.7%	122	53.3%	57	59.4%
26	100	52.6%	63	63.6%	112	48.9%	56	58.3%	26	107	56.3%	65	65.7%	122	53.3%	58	60.4%
27	100	52.6%	63	63.6%	112	48.9%	56	58.3%	27	107	56.3%	65	65.7%	122	53.3%	58	60.4%
28	101	53.2%	63	63.6%	114	49.8%	56	58.3%	28	107	56.3%	65	65.7%	122	53.3%	58	60.4%
29	101	53.2%	64	64.6%	114	49.8%	56	58.3%	29	107	56.3%	65	65.7%	122	53.3%	58	60.4%
30	101	53.2%	64	64.6%	114	49.8%	56	58.3%	30	107	56.3%	66	66.7%	122	53.3%	58	60.4%
31	101	53.2%	64	64.6%	114	49.8%	56	58.3%									

SARS-CoV-2 = severe acute respiratory syndrome coronavirus 2, , P4 = period 4 (March 1, 2022 to April 30, 2022), Data are cumulative numbers (No.) and percentages (%) of SARS-CoV-2 infections detected, No. = Number, % = Percentage, Mo. = month, M.S. = middle school (Students aged 13.1 ± 1.3 years old), H.S. = 4-year high school (Students aged 16.9 ± 1.2 years old), GB = students in school classes with a general school branch, SF = students in school classes with sport focus, n = Study population, Mar.22 = March 2022, Apr.22 = April 2022.

Table S8. Detailed 7-day incidences for detected SARS-CoV-2 infections for P1 (Sep.21 and Oct.21).

Mo.	M.S.		H.S.		AGES		Mo.	M.S.		H.S.		AGES	
Sep.21	GB (n=190)	SF (n=99)	GB (n=229)	SF (n=96)	I	II	Oct.21	GB (n=190)	SF (n=99)	GB (n=229)	SF (n=96)	I	II
1							1	0.0	1010.1	436.7	0.0	422.5	197.9
2							2	0.0	1010.1	436.7	0.0	410.7	203.4
3							3	0.0	1010.1	436.7	0.0	383.0	186.8
4							4	0.0	1010.1	0.0	0.0	363.3	172.0
5							5	0.0	1010.1	436.7	0.0	353.4	209.0
6							6	0.0	0.0	436.7	0.0	317.9	212.7
7							7	0.0	0.0	436.7	0.0	302.1	214.5
8							8	0.0	0.0	436.7	0.0	306.0	216.4
9							9	0.0	0.0	436.7	0.0	300.1	223.8
10							10	0.0	0.0	436.7	0.0	296.2	225.6
11							11	0.0	0.0	436.7	0.0	276.4	242.3
12							12	0.0	0.0	0.0	0.0	246.8	227.5
13	0.0	0.0	0.0	0.0	207.3	183.1	13	0.0	0.0	0.0	0.0	270.5	231.2
14	0.0	0.0	0.0	0.0	215.2	194.2	14	0.0	0.0	0.0	1041.7	266.5	240.4
15	0.0	0.0	0.0	0.0	256.7	214.5	15	0.0	0.0	0.0	1041.7	258.6	255.2
16	0.0	0.0	0.0	0.0	229.0	203.4	16	0.0	0.0	0.0	1041.7	246.8	260.8
17	0.0	0.0	0.0	0.0	240.9	210.8	17	0.0	0.0	0.0	1041.7	256.7	273.7
18	0.0	0.0	0.0	0.0	225.1	197.9	18	0.0	0.0	0.0	1041.7	284.3	275.6
19	0.0	0.0	0.0	0.0	211.3	184.9	19	0.0	0.0	0.0	1041.7	329.7	308.9
20	0.0	0.0	0.0	0.0	191.5	168.3	20	526.3	1010.1	436.7	1041.7	371.2	353.2
21	0.0	0.0	0.0	0.0	211.3	166.4	21	526.3	1010.1	436.7	0.0	402.8	336.6
22	0.0	0.0	0.0	0.0	201.4	144.3	22	1052.6	1010.1	436.7	0.0	438.3	371.7
23	0.0	0.0	0.0	1041.7	223.1	138.7	23	1052.6	1010.1	436.7	0.0	535.1	416.1
24	0.0	0.0	0.0	1041.7	217.2	135.0	24	1052.6	1010.1	436.7	0.0	556.8	419.8
25	0.0	0.0	0.0	1041.7	258.6	138.7	25	1052.6	1010.1	436.7	0.0	645.6	442.0
26	0.0	0.0	0.0	1041.7	294.2	162.7	26	1052.6	1010.1	436.7	0.0	695.0	445.7
27	0.0	0.0	436.7	1041.7	337.6	184.9	27	526.3	0.0	0.0	1041.7	671.3	455.0
28	0.0	0.0	436.7	1041.7	367.2	184.9	28	526.3	0.0	436.7	1041.7	750.3	543.7
29	0.0	1010.1	436.7	1041.7	400.8	199.7	29	0.0	0.0	436.7	1041.7	825.3	580.7
30	0.0	1010.1	436.7	0.0	422.5	203.4	30	0.0	0.0	436.7	1041.7	811.5	601.1
							31	0.0	0.0	436.7	1041.7	825.3	615.9

SARS-CoV-2 = severe acute respiratory syndrome coronavirus 2, P1 = period 1 (September 13, 2021 to October 31, 2021), Data are raw daily 7-day incidences extrapolated to 100,000 persons, Mo. = month, M.S. = middle school (students aged 13.1 ± 1.3 years old), H.S. = 4-year high school (students aged 16.9 ± 1.2 years old), AGES = Austrian Agency for Health and Food Safety, GB = students in school classes with a general school branch, SF = students in school classes with sport focus, I = 7 days incidence for SARS-CoV-2 infections per 100,000 inhabitants in Carinthia (AUT) for children aged 5 to 14 years (https://covid19-dashboard.ages.at/), II = 7 days incidence for SARS-CoV-2 infections per 100,000 inhabitants in Carinthia (AUT) for adolescent aged 15 to 24 years (https://covid19-dashboard.ages.at/), AUT = Austria, n = Study population, Sep.21 = September 2021, Oct.21 = October 2021.

Table S9. Detailed 7-day incidences for detected SARS-CoV-2 infections for P2 (Nov.21 and Dec.21).

Mo.	M.S.		H.S.		AGES		Mo.	M.S.		H.S.		AGES	
Nov.21	GB (n=190)	SF (n=99)	GB (n=229)	SF (n=96)	I	II	Dec.21	GB (n=190)	SF (n=99)	GB (n=229)	SF (n=96)	I	II
1	0.0	1010.1	436.7	1041.7	813.4	630.7	1	2105.3	6060.6	436.7	1041.7	3161.0	1387.1
2	0.0	2020.2	436.7	1041.7	795.7	634.4	2	1579.0	3030.3	436.7	1041.7	2657.5	1209.5
3	0.0	3030.3	436.7	1041.7	801.6	686.1	3	1052.6	1010.1	0.0	0.0	2221.2	1070.8
4	526.3	3030.3	436.7	1041.7	894.4	828.5	4	1052.6	1010.1	0.0	0.0	1842.1	934.0
5	526.3	3030.3	1310.0	2083.3	943.8	941.4	5	1052.6	1010.1	436.7	0.0	1642.7	832.2
6	526.3	3030.3	1310.0	3125.0	937.8	1050.5	6	1579.0	1010.1	873.4	0.0	1453.1	786.0
7	526.3	3030.3	1746.7	3125.0	959.5	1142.9	7	1052.6	1010.1	1310.0	0.0	1230.0	686.1
8	526.3	3030.3	2183.4	4166.7	973.4	1274.3	8	526.3	1010.1	1310.0	0.0	937.8	619.6
9	526.3	2020.2	2183.4	5208.3	1066.2	1355.6	9	526.3	1010.1	1310.0	0.0	764.1	541.9
10	526.3	1010.1	2620.1	5208.3	1551.9	1644.1	10	1052.6	1010.1	1746.7	0.0	856.9	562.2
11	0.0	1010.1	3056.8	5208.3	1555.8	1614.5	11	1579.0	1010.1	1746.7	0.0	740.4	525.2
12	526.3	1010.1	2183.4	4166.7	1561.7	1614.5	12	1579.0	1010.1	1310.0	0.0	738.4	530.8
13	1052.6	1010.1	2183.4	3125.0	1660.4	1551.7	13	1579.0	0.0	1746.7	0.0	661.4	471.6
14	1052.6	1010.1	2183.4	3125.0	1737.4	1548.0	14	1579.0	0.0	1310.0	0.0	576.5	408.7
15	1579.0	0.0	2183.4	3125.0	1891.4	1549.8	15	2105.3	0.0	1310.0	0.0	616.0	379.1
16	1579.0	1010.1	2183.4	2083.3	2084.9	1670.0	16	2105.3	0.0	1310.0	0.0	647.6	384.7
17	1579.0	1010.1	2183.4	1041.7	2460.1	1651.5	17	1579.0	0.0	873.4	0.0	501.5	329.2
18	1579.0	1010.1	1746.7	1041.7	2851.0	1832.8	18	1052.6	0.0	873.4	0.0	493.6	295.9
19	1052.6	1010.1	2183.4	2083.3	3054.4	1890.1	19	1052.6	0.0	873.4	0.0	466.0	260.8
20	526.3	1010.1	2183.4	4166.7	3324.8	2051.0	20	526.3	0.0	0.0	0.0	454.1	233.0
21	526.3	1010.1	1746.7	4166.7	3340.6	1997.4	21	526.3	0.0	0.0	0.0	446.2	227.5
22	0.0	1010.1	1310.0	3125.0	3372.2	1986.3	22	0.0	0.0	0.0	0.0	430.4	197.9
23	0.0	0.0	1310.0	3125.0	3295.2	1871.6	23	0.0	0.0	0.0	0.0	438.3	188.6
24	0.0	3030.3	873.4	3125.0	3228.1	1786.5	24	0.0	0.0	0.0	0.0	394.9	157.2
25	526.3	6060.6	436.7	3125.0	3279.4	1653.4	25	0.0	0.0	0.0	0.0	359.3	146.1
26	1052.6	8080.8	436.7	3125.0	3435.4	1585.0	26	0.0	1010.1	0.0	0.0	357.4	166.4
27	1052.6	8080.8	436.7	1041.7	3514.4	1505.4	27	0.0	1010.1	0.0	0.0	359.3	172.0
28	1052.6	8080.8	436.7	1041.7	3609.2	1572.0	28	0.0	1010.1	0.0	0.0	333.7	186.8
29	1052.6	9090.9	436.7	1041.7	3605.2	1527.6	29	0.0	2020.2	0.0	0.0	260.6	192.3
30	1579.0	9090.9	436.7	1041.7	3662.5	1551.7	30	0.0	2020.2	0.0	0.0	252.7	210.8
							31	0.0	2020.2	0.0	0.0	274.4	231.2

SARS-CoV-2 = severe acute respiratory syndrome coronavirus 2, P2 = period 2 (November 1, 2021 to December 31, 2021), Data are raw daily 7-day incidences extrapolated to 100,000 persons, Mo. = month, M.S. = middle school (students aged 13.1 ± 1.3 years old), H.S. = 4-year high school (students aged 16.9 ± 1.2 years old), AGES = Austrian Agency for Health and Food Safety, GB = students in school classes with a general school branch, SF = students in school classes with sport focus, I = 7 days incidence for SARS-CoV-2 infections per 100,000 inhabitants in Carinthia (AUT) for children aged 5 to 14 years (https://covid19-dashboard.ages.at/), II = 7 days incidence for SARS-CoV-2 infections per 100,000 inhabitants in Carinthia (AUT) for adolescent aged 15 to 24 years (https://covid19-dashboard.ages.at/), AUT = Austria, n = Study population, Nov.21 = November 2021, Dec.21 = December 2021.

Table S10. Detailed 7-day incidences for detected SARS-CoV-2 infections for P3 (Jan.22 and Feb.22).

Mo.	M.S.		H.S.		AGES		Mo.	M.S.		H.S.		AGES	
Jan.22	GB (n=190)	SF (n=99)	GB (n=229)	SF (n=96)	I	II	Feb.22	GB (n=190)	SF (n=99)	GB (n=229)	SF (n=96)	I	II
1	0.0	2020.2	436.7	1041.7	260.6	240.4	1	3684.2	11111.1	3930.1	7291.7	4282.4	3238.3
2	0.0	1010.1	436.7	1041.7	260.6	227.5	2	4210.5	11111.1	4366.8	8333.3	4207.4	3258.7
3	0.0	1010.1	436.7	2083.3	238.9	257.1	3	4736.8	8080.8	3930.1	6250.0	4262.7	3184.7
4	0.0	1010.1	436.7	2083.3	258.6	369.9	4	5263.2	8080.8	3930.1	7291.7	4641.8	3454.7
5	526.3	0.0	873.4	2083.3	292.2	519.7	5	5789.5	9090.9	4366.8	7291.7	4866.8	3711.8
6	526.3	0.0	1310.0	2083.3	321.8	654.7	6	5789.5	10101.0	3056.8	8333.3	5074.1	3904.1
7	526.3	1010.1	1746.7	2083.3	347.5	712.0	7	5263.2	8080.8	3056.8	5208.3	5127.4	3867.1
8	526.3	1010.1	1746.7	2083.3	408.7	865.5	8	4210.5	11111.1	2620.1	5208.3	5528.2	4218.5
9	1052.6	2020.2	2183.4	2083.3	456.1	950.6	9	4736.8	8080.8	3493.5	4166.7	5873.8	4446.0
10	3157.9	3030.3	2183.4	2083.3	586.4	1109.7	10	4210.5	11111.1	3930.1	5208.3	5794.8	4532.9
11	4210.5	4040.4	2183.4	3125.0	758.2	1213.2	11	3157.9	11111.1	3930.1	4166.7	5348.6	4270.3
12	3684.2	4040.4	1746.7	4166.7	807.5	1248.4	12	3684.2	10101.0	3056.8	4166.7	4924.1	4016.9
13	4210.5	4040.4	1310.0	5208.3	935.9	1294.6	13	4736.8	9090.9	3493.5	2083.3	4714.8	3885.6
14	4210.5	3030.3	873.4	5208.3	1028.6	1388.9	14	5263.2	8080.8	3493.5	2083.3	4507.5	3918.9
15	4210.5	3030.3	1746.7	6250.0	1129.3	1416.7	15	5263.2	6060.6	5240.2	1041.7	3753.3	3476.9
16	3684.2	2020.2	1310.0	10416.7	1239.9	1470.3	16	4210.5	5050.5	3930.1	1041.7	3016.8	3208.7
17	1579.0	2020.2	2620.1	10416.7	1342.6	1477.7	17	4736.8	2020.2	3493.5	0.0	2521.3	3001.6
18	1052.6	2020.2	3056.8	11458.3	1504.5	1631.2	18	5263.2	2020.2	4366.8	0.0	2270.5	2890.6
19	1579.0	2020.2	4366.8	13541.7	1836.2	1768.0	19	5789.5	2020.2	4366.8	1041.7	2246.8	2850.0
20	1052.6	3030.3	5240.2	14583.3	2071.1	1919.7	20	6842.1	2020.2	4366.8	1041.7	2260.7	2781.5
21	1579.0	5050.5	5676.9	18750.0	2631.8	2243.3	21	7368.4	3030.3	4366.8	1041.7	2321.9	2740.8
22	2105.3	6060.6	4366.8	19791.7	2825.3	2346.9	22	7894.7	2020.2	3493.5	1041.7	2689.1	2905.4
23	2631.6	7070.7	4366.8	16666.7	2983.3	2485.6	23	7894.7	3030.3	4803.5	1041.7	2935.9	2905.4
24	2631.6	7070.7	3056.8	15625.0	3082.0	2544.8	24	6315.8	3030.3	5676.9	1041.7	2977.4	2944.3
25	2631.6	6060.6	3056.8	14583.3	3482.8	2698.3	25	6842.1	2020.2	5240.2	1041.7	3208.4	3084.8
26	2105.3	10101.0	1746.7	11458.3	3816.5	2872.1	26	5263.2	3030.3	5240.2	0.0	3172.8	2977.6
27	3157.9	12121.2	1310.0	11458.3	4280.4	3158.8	27	3157.9	3030.3	4803.5	0.0	3149.1	2938.7
28	3157.9	11111.1	873.4	7291.7	4185.7	3105.2	28	2631.6	2020.2	4366.8	0.0	3101.7	2931.3
29	2631.6	10101.0	1310.0	4166.7	4302.2	3171.8							
30	2105.3	9090.9	2620.1	4166.7	4239.0	3155.1							
31	2631.6	11111.1	3493.5	7291.7	4339.7	3260.5							

SARS-CoV-2 = severe acute respiratory syndrome coronavirus 2, , P3 = period 3 (January 1, 2022 to February 28, 2022), Data are raw daily 7-day incidences extrapolated to 100,000 persons, Mo. = month, M.S. = middle school (students aged 13.1 ± 1.3 years old), H.S. = 4-year high school (students aged 16.9 ± 1.2 years old), AGES = Austrian Agency for Health and Food Safety, GB = students in school classes with a general school branch, SF = students in school classes with sport focus, I = 7 days incidence for SARS-CoV-2 infections per 100,000 inhabitants in Carinthia (AUT) for children aged 5 to 14 years (https://covid19-dashboard.ages.at/), II = 7 days incidence for SARS-CoV-2 infections per 100,000 inhabitants in Carinthia (AUT) for adolescent aged 15 to 24 years (https://covid19-dashboard.ages.at/), AUT = Austria, n = Study population, Jan.22 = January 2022, Feb.22 = February 2022.

Table S11. Detailed 7-day incidences for detected SARS-CoV-2 infections for P4 (Mar.22 and Apr.22).

Mo.	M.S.		H.S.		AGES		Mo.	M.S.		H.S.		AGES	
Mar.22	GB (n=190)	SF (n=99)	GB (n=229)	SF (n=96)	I	II	Apr.22	GB (n=190)	SF (n=99)	GB (n=229)	SF (n=96)	I	II
1	1579.0	3030.3	4803.5	0.0	3018.8	2877.7	1	1052.6	2020.2	1746.7	1041.7	1891.4	1686.7
2	2631.6	2020.2	3930.1	0.0	3403.8	3001.6	2	1052.6	2020.2	1746.7	1041.7	1842.1	1579.4
3	3157.9	2020.2	3056.8	0.0	3474.9	3058.9	3	2631.6	2020.2	1746.7	1041.7	1776.9	1475.8
4	2105.3	3030.3	3056.8	1041.7	3747.4	3138.5	4	2105.3	3030.3	873.4	1041.7	1660.4	1351.9
5	2105.3	2020.2	3930.1	1041.7	3861.9	3279.0	5	2105.3	2020.2	873.4	2083.3	1563.7	1270.6
6	2105.3	2020.2	3930.1	1041.7	3913.2	3297.5	6	2105.3	2020.2	1310.0	1041.7	1350.5	1091.2
7	1579.0	2020.2	3930.1	1041.7	4102.7	3373.3	7	2105.3	2020.2	1746.7	1041.7	1283.3	1000.5
8	2105.3	1010.1	2183.4	1041.7	4539.1	3669.2	8	2105.3	1010.1	1310.0	1041.7	1082.0	882.2
9	3157.9	1010.1	3056.8	1041.7	4477.9	3978.1	9	1579.0	1010.1	1310.0	1041.7	1056.3	869.2
10	3684.2	1010.1	4366.8	1041.7	4612.1	4266.6	10	526.3	1010.1	1310.0	1041.7	1024.7	832.2
11	5263.2	0.0	3930.1	0.0	4675.3	4488.5	11	526.3	0.0	1310.0	1041.7	981.3	795.3
12	6842.1	0.0	3056.8	0.0	4734.5	4625.4	12	526.3	0.0	1310.0	0.0	675.2	630.7
13	6842.1	0.0	3056.8	0.0	4805.6	4725.3	13	526.3	0.0	873.4	0.0	519.3	536.3
14	8421.1	0.0	2620.1	0.0	4740.5	4778.9	14	526.3	0.0	873.4	0.0	493.6	499.3
15	8947.4	0.0	3493.5	0.0	4647.7	4817.7	15	1052.6	0.0	436.7	0.0	351.4	427.2
16	8947.4	0.0	4366.8	0.0	4849.1	4814.0	16	1052.6	0.0	436.7	0.0	333.7	386.5
17	8421.1	0.0	3493.5	0.0	4701.0	4630.9	17	526.3	0.0	436.7	1041.7	319.8	368.0
18	7368.4	0.0	4366.8	0.0	4651.6	4514.4	18	526.3	0.0	436.7	1041.7	315.9	364.3
19	5789.5	0.0	4366.8	0.0	4556.9	4396.1	19	526.3	0.0	873.4	1041.7	331.7	331.0
20	5789.5	0.0	6113.5	0.0	4432.5	4360.9	20	526.3	0.0	1310.0	1041.7	475.8	408.7
21	4736.8	1010.1	7423.6	0.0	4306.1	4296.2	21	526.3	0.0	873.4	1041.7	467.9	412.4
22	4210.5	1010.1	7860.3	0.0	4183.7	4081.7	22	0.0	0.0	873.4	1041.7	489.6	432.8
23	2105.3	2020.2	6550.2	0.0	3761.2	3650.8	23	0.0	0.0	873.4	2083.3	483.7	421.7
24	2105.3	2020.2	6113.5	0.0	3696.0	3382.6	24	0.0	0.0	873.4	1041.7	481.7	432.8
25	1579.0	2020.2	5240.2	0.0	3194.5	3040.4	25	0.0	0.0	1310.0	1041.7	501.5	427.2
26	2105.3	2020.2	5240.2	0.0	3034.6	2861.1	26	0.0	0.0	873.4	1041.7	517.3	440.2
27	2105.3	2020.2	3493.5	0.0	2890.5	2718.6	27	0.0	0.0	436.7	1041.7	404.7	356.9
28	2105.3	1010.1	3056.8	0.0	2787.8	2537.4	28	0.0	0.0	436.7	1041.7	430.4	351.4
29	1579.0	2020.2	1746.7	0.0	2351.5	2171.2	29	0.0	0.0	436.7	1041.7	424.5	318.1
30	1579.0	1010.1	873.4	1041.7	2100.7	1997.4	30	0.0	1010.1	436.7	0.0	446.2	303.3
31	1052.6	1010.1	873.4	1041.7	1838.1	1784.7							

SARS-CoV-2 = severe acute respiratory syndrome coronavirus 2, , P4 = period 4 (March 1, 2022 to April 30, 2022), Data are raw daily 7-day incidences extrapolated to 100,000 persons, Mo. = month, M.S. = middle school (students aged 13.1 ± 1.3 years old), H.S. = 4-year high school (students aged 16.9 ± 1.2 years old), AGES = Austrian Agency for Health and Food Safety, GB = students in school classes with a general school branch, SF = students in school classes with sport focus, I = 7 days incidence for SARS-CoV-2 infections per 100,000 inhabitants in Carinthia (AUT) for children aged 5 to 14 years (https://covid19-dashboard.ages.at/), II = 7 days incidence for SARS-CoV-2 infections per 100,000 inhabitants in Carinthia (AUT) for adolescent aged 15 to 24 years (https://covid19-dashboard.ages.at/), AUT = Austria, n = Study population, Mar.22 = March 2022, Apr.22 = April 2022.

Table S12. Potential SARS-CoV-2 infection in school classes GB vs. SF classes, using hypothesized generation time - 2 days.

Time Period	school grade	Category	HTPPI = GT 2D						
			PI, No.		X^2	P Value [b]	p-lvl	φ	OR (95%CI)
			yes	no					
P1	All (n=614)	GB (n=419)	0	419	INC				
		SF (n=185)	0	195					
	M.S. (n=289)	GB (n=190)	0	190	INC				
		SF (n=99)	0	99					
	H.S. (n=325)	GB (n=229)	0	229	INC				
		SF (n=96)	0	96					
P2	All (n=614)	GB (n=419)	3	416	6.859 [a]	0.014	*	0.11	5.16 (1.32 to 20.19)
		SF (n=185)	7	188					
	M.S. (n=289)	GB (n=190)	0	190	INC				
		SF (n=99)	5	94					
	H.S. (n=325)	GB (n=229)	3	226	0.267 [a]	0.63		0.03	1.60 (0.26 to 9.75)
		SF (n=96)	2	94					
P3	All (n=614)	GB (n=419)	26	393	30.066	<.001	***	0.22	4.02 (2.38 to 6.81)
		SF (n=185)	41	154					
	M.S. (n=289)	GB (n=190)	12	178	15.864	<.001	***	0.23	4.24 (2.00 to 8.99)
		SF (n=99)	22	77					
	H.S. (n=325)	GB (n=229)	14	215	13.872	<.001	***	0.21	3.79 (1.81 to 7.93)
		SF (n=96)	19	77					
P4	All (n=614)	GB (n=419)	28	391	INC				
		SF (n=185)	0	195					
	M.S. (n=289)	GB (n=190)	14	176	INC				
		SF (n=99)	0	99					
	H.S. (n=325)	GB (n=229)	14	215	INC				
		SF (n=96)	0	96					

a = 1 cell (25%) or 2 cells (50,0%) have expected count less than 5; b = If (a) occurred, Fisher's Exact Test was used. SARS-CoV-2 = severe acute respiratory syndrome coronavirus 2, HTPPI = hypothetical time period assumed for potential infections, GT = generation time, D = days, PI = potential infection, No = absolute Number of children, X^2 = Chi-Square Test value, φ = Effect size Phi , p-lvl (P Value level) * = P < 0.05, ** = P < 0.01, *** = P < 0.001, p-lvl = significance level, OR = Odds ratio for potential SARS-CoV-2 infection in school classes with sport focus vs. school classes without sport focus, using different hypothesized generation time (with 1 for students in classes without sport focus), CI = Confidence Interval, P1 = period 1 (September 13, 2021 to October 31, 2021) P2 = period 2 (November 1, 2021 to December 31, 2021), P3 = period 3 (January 1, 2022 to February 28, 2022), P4 = period 4 (March 1, 2022 to April 30, 2022), M.S. = middle school (students aged 13.1 ± 1.3 years old), H.S. = 4-year high school (students aged 16.9 ± 1.2 years old), GB = students in school classes with a general school branch, SF = students in school classes with sport focus, n = Study population, INC = insufficient number of potential infection cases.

Table S13. Potential SARS-CoV-2 infection in school classes GB vs. SF classes, using hypothesized generation time - 4 days.

Time Period	school grade	Category	HTPPI = GT 4D						
			PI, No.		X^2	P Value [b]	p-lvl	φ	OR (95%CI)
			yes	no					
P1	All (n=614)	GB (n=419)	0	419	INC				
		SF (n=185)	0	195					
	M.S. (n=289)	GB (n=190)	0	190	INC				
		SF (n=99)	0	99					
	H.S. (n=325)	GB (n=229)	0	229	INC				
		SF (n=96)	0	96					
P2	All (n=614)	GB (n=419)	8	411	4.845	0.028	*	0.09	2.78 (1.08 to 7.15)
		SF (n=185)	10	185					
	M.S. (n=289)	GB (n=190)	1	189	8.435 [a]	0.007	**	0.17	12.19 (1.45 to 102.76)
		SF (n=99)	6	93					
	H.S. (n=325)	GB (n=229)	7	222	0.255 [a]	0.74		0.03	1.38 (0.39 to 4.82)
		SF (n=96)	4	92					
P3	All (n=614)	GB (n=419)	39	380	40.042	<.001	***	0.26	4.03 (2.56 to 6.31)
		SF (n=185)	57	138					
	M.S. (n=289)	GB (n=190)	18	172	17.205	<.001	***	0.24	3.77 (1.96 to 7.24)
		SF (n=99)	28	71					
	H.S. (n=325)	GB (n=229)	21	208	22.998	<.001	***	0.27	4.29 (2.29 to 8.01)
		SF (n=96)	29	67					
P4	All (n=614)	GB (n=419)	37	382	15.857	<.001	***	-0.16	0.05 (0.01 to 0.39)
		SF (n=185)	1	194					
	M.S. (n=289)	GB (n=190)	17	173	7.021	0.008	**	-0.16	0.10 (0.01 to 0.79)
		SF (n=99)	1	98					
	H.S. (n=325)	GB (n=229)	20	209	8.934	0.003	**	-0.16	0.10 (0.01 to 0.79)
		SF (n=96)	0	96					

a = 1 cell (25%) or 2 cells (50,0%) have expected count less than 5; b = If (a) occurred, Fisher's Exact Test was used.
SARS-CoV-2 = severe acute respiratory syndrome coronavirus 2, HTPPI = hypothetical time period assumed for potential infections, GT = generation time, D = days, PI = potential infection, No = absolute Number of children, X^2 = Chi-Square Test value, φ = Effect size Phi , p-lvl (P Value level) * = P < 0.05, ** = P < 0.01, *** = P < 0.001, p-lvl = significance level, OR = Odds ratio for potential SARS-CoV-2 infection in school classes with sport focus vs. school classes without sport focus, using different hypothesized generation time (with 1 for students in classes without sport focus), CI = Confidence Interval, P1 = period 1 (September 13, 2021 to October 31, 2021) P2 = period 2 (November 1, 2021 to December 31, 2021), P3 = period 3 (January 1, 2022 to February 28, 2022), P4 = period 4 (March 1, 2022 to April 30, 2022), M.S. = middle school (students aged 13.1 ± 1.3 years old), H.S. = 4-year high school (students aged 16.9 ± 1.2 years old), GB = students in school classes with a general school branch, SF = students in school classes with sport focus, n = Study population, INC = insufficient number of potential infection cases.

Table S14. Potential SARS-CoV-2 infection in school classes GB vs. SF classes, using hypothesized generation time - 6 days.

Time Period	school grade	Category	HTPPI = GT 6D							
			PI, No.		X^2	P Value [b]	p-lvl	φ	OR (95%CI)
			yes	no					
P1	All (n=614)	GB (n=419)	0	419	INC				
		SF (n=185)	0	195					
	M.S. (n=289)	GB (n=190)	0	190	INC				
		SF (n=99)	0	99					
	H.S. (n=325)	GB (n=229)	0	229	INC				
		SF (n=96)	0	96					
P2	All (n=614)	GB (n=419)	10	409	6.761	0.009	**	0.11	2.92 (1.26 to 6.79)
		SF (n=185)	13	182					
	M.S. (n=289)	GB (n=190)	3	187	4.333 [a]	0.07		0.04	4.02 (0.98 to 16.44)
		SF (n=99)	6	93					
	H.S. (n=325)	GB (n=229)	7	222	2.943 [a]	0.13		0.09	2.49 (0.85 to 7.32)
		SF (n=96)	7	89					
P3	All (n=614)	GB (n=419)	45	374	39.313	<.001	***	0.25	3.78 (2.45 to 5.83)
		SF (n=185)	61	134					
	M.S. (n=289)	GB (n=190)	21	169	16.597	<.001	***	0.24	3.50 (1.88 to 6.53)
		SF (n=99)	30	69					
	H.S. (n=325)	GB (n=229)	24	205	22.889	<.001	***	0.27	4.08 (2.23 to 7.43)
		SF (n=96)	31	65					
P4	All (n=614)	GB (n=419)	51	368	18.758	<.001	***	-0.175	0.11 (0.04 to 0.37)
		SF (n=185)	3	192					
	M.S. (n=289)	GB (n=190)	23	167	8.377	0.004	**	-0.17	0.15 (0.04 to 0.65)
		SF (n=99)	2	97					
	H.S. (n=325)	GB (n=229)	28	201	10.414	0.001	**	-0.18	0.08 (0.01 to 0.56)
		SF (n=96)	1	95					

a = 1 cell (25%) or 2 cells (50,0%) have expected count less than 5; b = If (a) occurred, Fisher's Exact Test was used. SARS-CoV-2 = severe acute respiratory syndrome coronavirus 2, HTPPI = hypothetical time period assumed for potential infections, GT = generation time, D = days, PI = potential infection, No = absolute Number of children, X^2 = Chi-Square Test value, φ = Effect size Phi , p-lvl (P Value level) * = P < 0.05, ** = P < 0.01, *** = P < 0.001, p-lvl = significance level, OR = Odds ratio for potential SARS-CoV-2 infection in school classes with sport focus vs. school classes without sport focus, using different hypothesized generation time (with 1 for students in classes without sport focus), CI = Confidence Interval, P1 = period 1 (September 13, 2021 to October 31, 2021) P2 = period 2 (November 1, 2021 to December 31, 2021), P3 = period 3 (January 1, 2022 to February 28, 2022), P4 = period 4 (March 1, 2022 to April 30, 2022), M.S. = middle school (students aged 13.1 ± 1.3 years old), H.S. = 4-year high school (students aged 16.9 ± 1.2 years old), GB = students in school classes with a general school branch, SF = students in school classes with sport focus, n = Study population, INC = insufficient number of potential infection cases.

Table S15. Potential SARS-CoV-2 infection in school classes GB vs. SF classes, using hypothesized generation time - 8 days.

Time Period	school grade	Category	HTPPI = GT 8D						
			PI, No.		X^2	P Value [b]	p-lvl	φ	OR (95%CI)
			yes	no					
P1	All (n=614)	GB (n=419)	2	417	INC				
		SF (n=185)	0	195					
	M.S. (n=289)	GB (n=190)	0	190	INC				
		SF (n=99)	0	99					
	H.S. (n=325)	GB (n=229)	2	227	INC				
		SF (n=96)	0	96					
P2	All (n=614)	GB (n=419)	11	408	7.066	0.008	**	0.11	2.87 (1.28 to 6.44)
		SF (n=185)	14	181					
	M.S. (n=289)	GB (n=190)	3	187	5.876 [a]	0.035	*	0.14	4.74 (1.20 to 18.76)
		SF (n=99)	7	92					
	H.S. (n=325)	GB (n=229)	8	221	2.217 [a]	0.15		0.08	2.17 (0.77 to 6.17)
		SF (n=96)	7	89					
P3	All (n=614)	GB (n=419)	54	365	34.055	<.001	***	0.24	3.30 (2.18 to 4.99)
		SF (n=185)	64	131					
	M.S. (n=289)	GB (n=190)	24	166	13.377	<.001	***	0.22	3.01 (1.64 to 5.51)
		SF (n=99)	30	69					
	H.S. (n=325)	GB (n=229)	30	199	21.302	<.001	***	0.26	3.64 (2.06 to 6.42)
		SF (n=96)	34	62					
P4	All (n=614)	GB (n=419)	56	363	19.319	<.001	***	-0.18	0.14 (0.05 to 0.38)
		SF (n=185)	4	191					
	M.S. (n=289)	GB (n=190)	25	165	7.630	0.006	**	-0.16	0.21 (0.06 to 0.70)
		SF (n=99)	3	96					
	H.S. (n=325)	GB (n=229)	31	198	11.898	0.001	**	-0.19	0.07 (0.01 to 0.50)
		SF (n=96)	1	95					

a = 1 cell (25%) or 2 cells (50,0%) have expected count less than 5; b = If (a) occurred, Fisher's Exact Test was used. SARS-CoV-2 = severe acute respiratory syndrome coronavirus 2, HTPPI = hypothetical time period assumed for potential infections, GT = generation time, D = days, PI = potential infection, No = absolute Number of children, X^2 = Chi-Square Test value, φ = Effect size Phi , p-lvl (P Value level) * = P < 0.05, ** = P < 0.01, *** = P < 0.001, p-lvl = significance level, OR = Odds ratio for potential SARS-CoV-2 infection in school classes with sport focus vs. school classes without sport focus, using different hypothesized generation time (with 1 for students in classes without sport focus), CI = Confidence Interval, P1 = period 1 (September 13, 2021 to October 31, 2021) P2 = period 2 (November 1, 2021 to December 31, 2021), P3 = period 3 (January 1, 2022 to February 28, 2022), P4 = period 4 (March 1, 2022 to April 30, 2022), M.S. = middle school (students aged 13.1 ± 1.3 years old), H.S. = 4-year high school (students aged 16.9 ± 1.2 years old), GB = students in school classes with a general school branch, SF = students in school classes with sport focus, n = Study population, INC = insufficient number of potential infection cases.

Table S16. Binary logistic regression for Potential SARS-Cov-2 contagions in the classroom and generation times 4 and 6, for subgroup sex .

Time Period	school grade	Category	Sex	HTPPI = GT 4D			HTPPI = GT 6D		
				PI		OR (95%CI)	PI		OR (95%CI)
				yes	no		yes	no	
P1	All (n=614)	♂ (n=349)	A (n=203)	0	203	INC	0	203	INC
			B (n=146)	0	146		0	146	
		♀ (n=265)	A (n=216)	0	216	INC	0	216	INC
			B (n=49)	0	49		0	49	
	M.S. (n=289)	♂ (n=178)	A (n=106)	0	106	INC	0	106	INC
			B (n=72)	0	72		0	72	
		♀ (n=111)	A (n=84)	0	84	INC	0	84	INC
			B (n=27)	0	27		0	27	
	H.S. (n=325)	♂ (n=171)	A (n=97)	0	97	INC	0	97	INC
			B (n=74)	0	74		0	74	
		♀ (n=154)	A (n=132)	0	132	INC	0	132	INC
			B (n=22)	0	22		0	22	
P2	All (n=614)	♂ (n=349)	A (n=203)	4	199	3.66 (1.12 to 11.90)	5	198	3.22 (1.10 to 9.50)
			B (n=146)	10	136		11	135	
		♀ (n=265)	A (n=216)	4	212	0.81 (0.77 to 0.86)	5	211	1.15 (0.34 to 9.54)
			B (n=49)	0	49		2	47	
	M.S. (n=289)	♂ (n=178)	A (n=106)	1	105	9.55 (1.12 to 81.07)	2	104	4.73 (0.93 to 24.12)
			B (n=72)	6	66		6	66	
		♀ (n=111)	A (n=84)	0	84	INC	1	83	INC
			B (n=27)	0	27		0	27	
	H.S. (n=325)	♂ (n=171)	A (n=97)	3	94	1.79 (0.39 to 8.26)	3	94	2.27 (0.53 to 9.82)
			B (n=74)	4	70		5	69	
		♀ (n=154)	A (n=132)	4	128	0.85 (0.80 to 0.91)	4	128	3.20 (0.55 to 18.63)
			B (n=22)	0	22		2	20	
P3	All (n=614)	♂ (n=349)	A (n=203)	14	189	4.58 (2.37 to 8.85)	18	185	3.88 (2.12 to 7.11)
			B (n=146)	37	109		40	106	
		♀ (n=265)	A (n=216)	25	191	5.27 (2.60 to 10.67)	27	189	5.25 (2.62 to 10.52)
			B (n=49)	20	29		21	28	
	M.S. (n=289)	♂ (n=178)	A (n=106)	8	98	3.50 (1.41 to 8.70)	10	96	2.97 (1.27 to 6.93)
			B (n=72)	16	56		17	55	
		♀ (n=111)	A (n=84)	10	74	5.92 (2.17 to 16.19)	11	73	6.16 (2.30 to 16.51)
			B (n=27)	12	15		13	14	
	H.S. (n=325)	♂ (n=171)	A (n=97)	6	91	6.01 (2.28 to 15.83)	8	89	5.02 (2.09 to 12.04)
			B (n=74)	21	53		23	51	
		♀ (n=154)	A (n=132)	15	117	4.46 (1.61 to 12.38)	16	116	4.14 (1.50 to 11.42)
			B (n=22)	8	14		8	14	
P4	All (n=614)	♂ (n=349)	A (n=203)	14	189	0.56 (0.51 to 0.62)	21	182	0.12 (0.03 to 0.52)
			B (n=146)	0	146		2	144	
		♀ (n=265)	A (n=216)	23	193	0.18 (0.02 to 1.33)	30	186	0.13 (0.02 to 0.97)
			B (n=49)	1	48		1	48	
	M.S. (n=289)	♂ (n=178)	A (n=106)	8	98	0.58 (0.51 to 0.66)	11	95	0.12 (0.02 to 0.96)
			B (n=72)	0	72		1	71	
		♀ (n=111)	A (n=84)	9	75	0.32 (0.04 to 2.65)	12	72	0.23 (0.03 to 1.86)
			B (n=27)	1	26		1	26	
	H.S. (n=325)	♂ (n=171)	A (n=97)	6	91	0.55 (0.48 to 0.63)	10	87	0.12 (0.02 to 0.95)
			B (n=74)	0	74		1	73	
		♀ (n=154)	A (n=132)	14	118	INC	14	118	INC
			B (n=22)	0	22		0	22	

SARS-CoV-2 = severe acute respiratory syndrome coronavirus 2, HTPPI = hypothetical time period assumed for potential infections, GT = generation time, D = days, PI = potential infection, No = absolute Number of children, OR = Odds ratio for potential SARS-CoV-2 infection in school classes with sport focus vs. school classes without sport focus, using different hypothesized generation time (with 1 for students in classes without sport focus), CI = Confidence Interval, ♂ = Boys, ♀ = Girls, P1 = period 1 (September 13, 2021 to October 31, 2021) P2 = period 2 (November 1, 2021 to December 31, 2021), P3 = period 3 (January 1, 2022 to February 28, 2022), P4 = period 4 (March 1, 2022 to April 30, 2022), M.S. = middle school (students aged 13.1 ± 1.3 years old), H.S. = 4-year high school (students aged 16.9 ± 1.2 years old), GB = students in school classes with a general school branch, SF = students in school classes with sport focus, n = Study population, INC = insufficient number of potential infection cases.

Table S17. Binary logistic regression for Potential SARS-Cov-2 contagions in the classroom and generation times 2 and 8, for subgroup sex .

Time Period	school grade	Category	Sex	HTPPI = GT 2D			HTPPI = GT 8D		
				PI		OR (95%CI)	PI		OR (95%CI)
				yes	no		yes	no	
P1	All (n=614)	♂ (n=349)	A (n=203)	0	203	INC	1	202	INC
			B (n=146)	0	146		0	146	
		♀ (n=265)	A (n=216)	0	216	INC	1	215	INC
			B (n=49)	0	49		0	49	
	M.S. (n=289)	♂ (n=178)	A (n=106)	0	106	INC	0	106	INC
			B (n=72)	0	72		0	72	
		♀ (n=111)	A (n=84)	0	84	INC	0	84	INC
			B (n=27)	0	27		0	27	
	H.S. (n=325)	♂ (n=171)	A (n=97)	0	97	INC	1	96	INC
			B (n=74)	0	74		0	74	
		♀ (n=154)	A (n=132)	0	132	INC	1	131	INC
			B (n=22)	0	22		0	22	
P2	All (n=614)	♂ (n=349)	A (n=203)	2	201	5.06 (1.04 to 24.73)	5	198	3.55 (1.22 to 10.30)
			B (n=146)	7	139		12	135	
		♀ (n=265)	A (n=216)	1	215	INC	6	210	1.49 (0.30 to 7.61)
			B (n=49)	0	49		2	47	
	M.S. (n=289)	♂ (n=178)	A (n=106)	0	106	INC	2	104	5.60 (1.13 to 27.79)
			B (n=72)	5	67		7	65	
		♀ (n=111)	A (n=84)	0	84	INC	1	83	INC
			B (n=27)	0	27		0	27	
	H.S. (n=325)	♂ (n=171)	A (n=97)	2	95	1.32 (0.18 to 9.59)	3	94	2.27 (0.53 to 9.82)
			B (n=74)	2	72		5	69	
		♀ (n=154)	A (n=132)	1	131	INC	5	127	2.54 (0.46 to 13.99)
			B (n=22)	0	22		2	20	
P3	All (n=614)	♂ (n=349)	A (n=203)	9	194	4.45 (2.01 to 9.86)	21	182	3.50 (1.97 to 6.23)
			B (n=146)	25	121		42	104	
		♀ (n=265)	A (n=216)	17	199	5.68 (2.61 to 12.33)	33	183	4.52 (2.30 to 8.87)
			B (n=49)	16	33		22	27	
	M.S. (n=289)	♂ (n=178)	A (n=106)	6	100	3.67 (1.33 to 10.18)	11	95	2.67 (1.17 to 6.11)
			B (n=72)	13	59		17	55	
		♀ (n=111)	A (n=84)	6	78	6.50 (2.05 to 20.59)	13	71	5.07 (1.94 to 13.23)
			B (n=27)	9	18		13	14	
	H.S. (n=325)	♂ (n=171)	A (n=97)	3	94	6.07 (1.64 to 22.37)	10	87	4.44 (1.97 to 10.00)
			B (n=74)	12	62		25	49	
		♀ (n=154)	A (n=132)	11	121	5.13 (1.73 to 15.25)	20	112	3.88 (1.46 to 10.27)
			B (n=22)	7	15		9	13	
P4	All (n=614)	♂ (n=349)	A (n=203)	11	192	INC	25	178	0.15 (0.04 to 0.51)
			B (n=146)	0	146		3	143	
		♀ (n=265)	A (n=216)	17	199	INC	31	185	0.12 (0.02 to 0.93)
			B (n=49)	0	49		1	48	
	M.S. (n=289)	♂ (n=178)	A (n=106)	6	100	INC	13	93	0.20 (0.05 to 0.94)
			B (n=72)	0	72		2	72	
		♀ (n=111)	A (n=84)	8	76	INC	12	72	0.23 (0.03 to 1.86)
			B (n=27)	0	27		1	26	
	H.S. (n=325)	♂ (n=171)	A (n=97)	5	92	INC	12	85	0.10 (0.01 to 0.76)
			B (n=74)	0	74		1	73	
		♀ (n=154)	A (n=132)	9	123	INC	19	113	INC
			B (n=22)	0	22		0	22	

SARS-CoV-2 = severe acute respiratory syndrome coronavirus 2, HTPPI = hypothetical time period assumed for potential infections, GT = generation time, D = days, PI = potential infection, No = absolute Number of children, OR = Odds ratio for potential SARS-CoV-2 infection in school classes with sport focus vs. school classes without sport focus, using different hypothesized generation time (with 1 for students in classes without sport focus), CI = Confidence Interval, ♂ = Boys, ♀ = Girls, P1 = period 1 (September 13, 2021 to October 31, 2021) P2 = period 2 (November 1, 2021 to December 31, 2021), P3 = period 3 (January 1, 2022 to February 28, 2022), P4 = period 4 (March 1, 2022 to April 30, 2022), M.S. = middle school (students aged 13.1 ± 1.3 years old), H.S. = 4-year high school (students aged 16.9 ± 1.2 years old), GB = students in school classes with a general school branch, SF = students in school classes with sport focus, n = Study population, INC = insufficient number of potential infection cases.

Figure S1. Cumulative percentage of children with SARS-CoV-2 infections, for subgroup sex

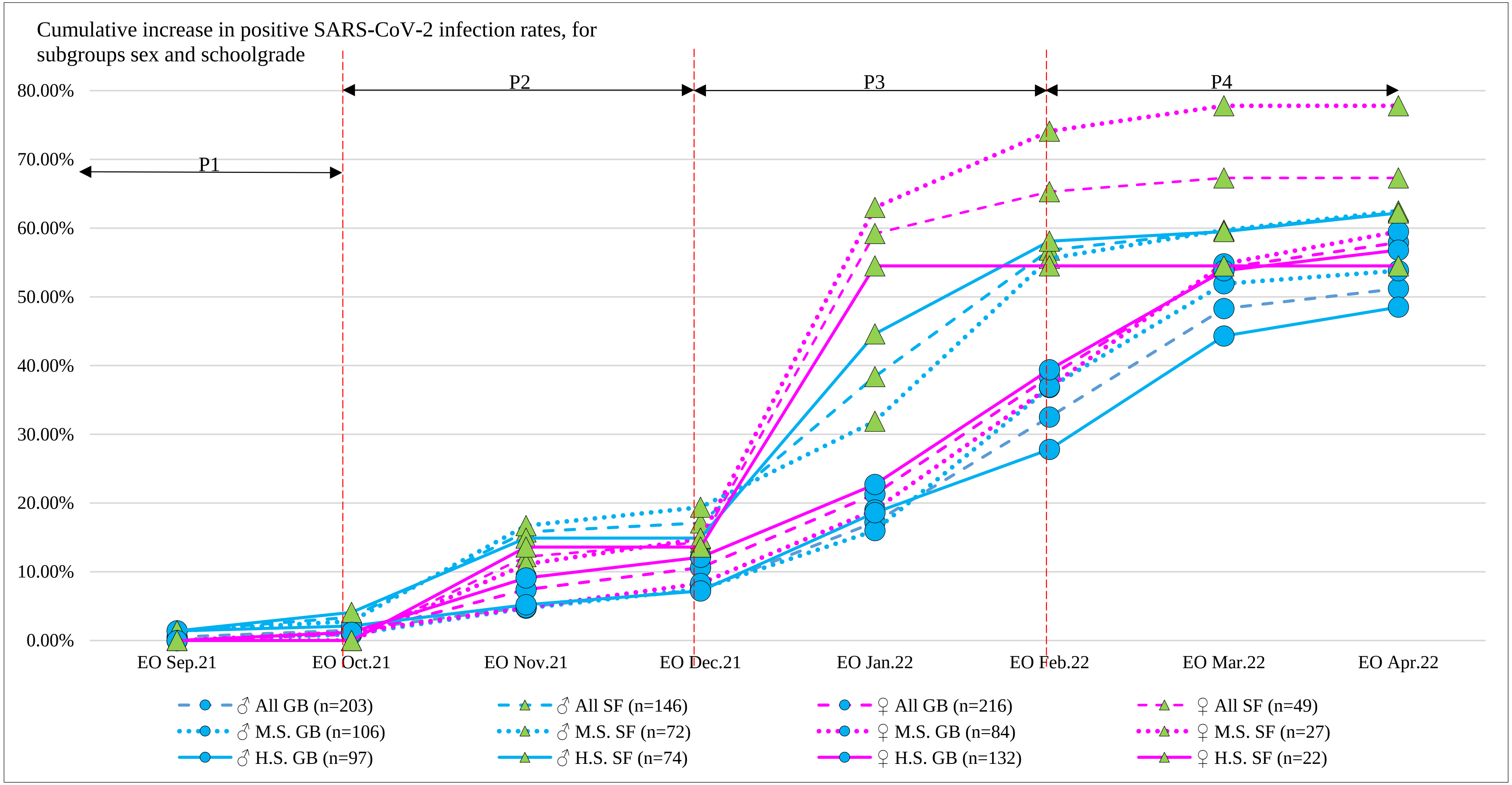

SARS-CoV-2 = severe acute respiratory syndrome coronavirus 2, P1 = period 1 (13. September to 31. October 2021), P1 = period 1 (September 13, 2021 to October 31, 2021) P2 = period 2 (November 1, 2021 to December 31, 2021), P3 = period 3 (January 1, 2022 to February 28, 2022), P4 = period 4 (March 1, 2022 to April 30, 2022), EO = End of, Sep.21 = September 2021, Oct. = October 2021, Nov.21 = November 2021, Dec.21 = December 2021, Jan.22 = January 2022, Feb.22 = February 2022, Mar.22 = March 2022, Apr.22 = April 2022, ♀ = Girls, ♂ = Boys, GB = students in school classes with a general school branch, SF = students in school classes with sport focus, M.S. = middle school (students aged 13.1 ± 1.3 years old), H.S. = 4-year high school (students aged 16.9 ± 1.2 years old), n = Study population.
